# Supplementary material for: CRISPRi gene modulation and all-optical electrophysiology in post-differentiated human iPSC-cardiomyocytes
Source: Commun Biol. 2023 Dec 7;6:1236. doi: 10.1038/s42003-023-05627-y (PMC10703822; doi:10.1038/s42003-023-05627-y)
Supplement: Supplementary file 1 — Supplemental Information [file 42003_2023_5627_MOESM1_ESM.pdf]

SUPPLEMENTARY INFORMATION

for

CRISPRi Gene Modulation and All-Optical Electrophysiology in Post-Differentiated Human iPSC-Cardiomyocytes

by Julie L. Han[1], Yuli W. Heinson[1], Christianne J. Chua[1], Wei Liu[1], Emilia Entcheva[1]

[1]Department of Biomedical Engineering, The George Washington University, Washington DC 20052, USA

Supplementary Table 1. Cell lines tested.

CELL TYPE	SEX	Tissue Source	AGE	SOURCE	IDENTIFIER
iCell[2]	Female	Fibroblast	18yr	FujiFilm/Cellular Dynamics	01434
iCell Diversity Panel	Female	N/A	50-59yr	FujiFilm/Cellular Dynamics	iPSC-CM-1X-01064
iCell Diversity Panel	Male	N/A	50-59yr	FujiFilm/Cellular Dynamics	IPSC-CM-1X-01063

Supplementary Table 2. gRNA oligo sequences tested, related to Methods.

gRNA Name	Oligo Sequence	Source
KCNH2 ERA1	GGGCTGCTCTGGTTGCCAGT	CRISPR-ERA
KCNH2 ERA2	GTTGCCAGTCGGCCAGCCTC	CRISPR-ERA
KCNH2 175	TTCTGGGCGCGCGAGTCCCA	HERG g–175 is a commonly used gRNA for efficient HERG knockdown in iPSC and iPSC-CM.
KCNH2 4112	CTCGATGTCGTCGGCCGACG	VectorBuilder
KCNH2 4113	CTCGTCGGCCGACGACATCG	VectorBuilder
KCNH2 4268	TAATGGTGCGGTAGCGCACG	VectorBuilder
KCNH2 5761	CGCGTTCATGTCGATGCCGT	VectorBuilder
KCNJ2 ERA1	CACACCGGCAGCGAGCGAAA	CRISPR-ERA
KCNJ2 ERA2	ATTCCCAAGACCCAGCCCGC	CRISPR-ERA
KCNJ2 736	AGACATCTTCACCACGTGTG	VectorBuilder
KCNJ2 777	TTTCATCATTGGCGCAGTCA	VectorBuilder
KCNJ2 787	TGTGGCGAGTGGGCAATCTT	VectorBuilder
GJA1 ERA1	TTCAAGCCACTGACTCAACT	CRISPR-ERA
GJA1 ERA2	TTCATTAGGGGGAAGGCGTG	CRISPR-ERA
GJA1 1215	GGGCGTTAAGGATCGGGTTA	VectorBuilder
GJA1 1218	CACCACTGGTCGCATGGTAA	VectorBuilder
GJA1 1219	GCACCACTGGTCGCATGGTA	VectorBuilder

Supplementary Table 3. Primers used for qPCR Analysis.

**qPCR**	**Oligo Sequence**<br>5' – Forward Primer – 3'<br>5' – Reverse Primer – 3'	**Source**
KCNH2	TCAACTGCGAGATACCAACATG<br>CTGGCTGCTCCGTGTCCTT	(Izumi-Nakaseko et al. 2017)
KCNJ2	GTGCGAACCAACCGCTACA<br>CCAGCGAATGTCCACACAC	
GJA1	GGTGACTGGAGCGCCTTAG<br>GCGCACATGAGAGATTGGGA	
GAPDH	GGAGCGAGATCCCTCCAAAAT<br>GGCTGTTGTCATACTTCTCATGG	

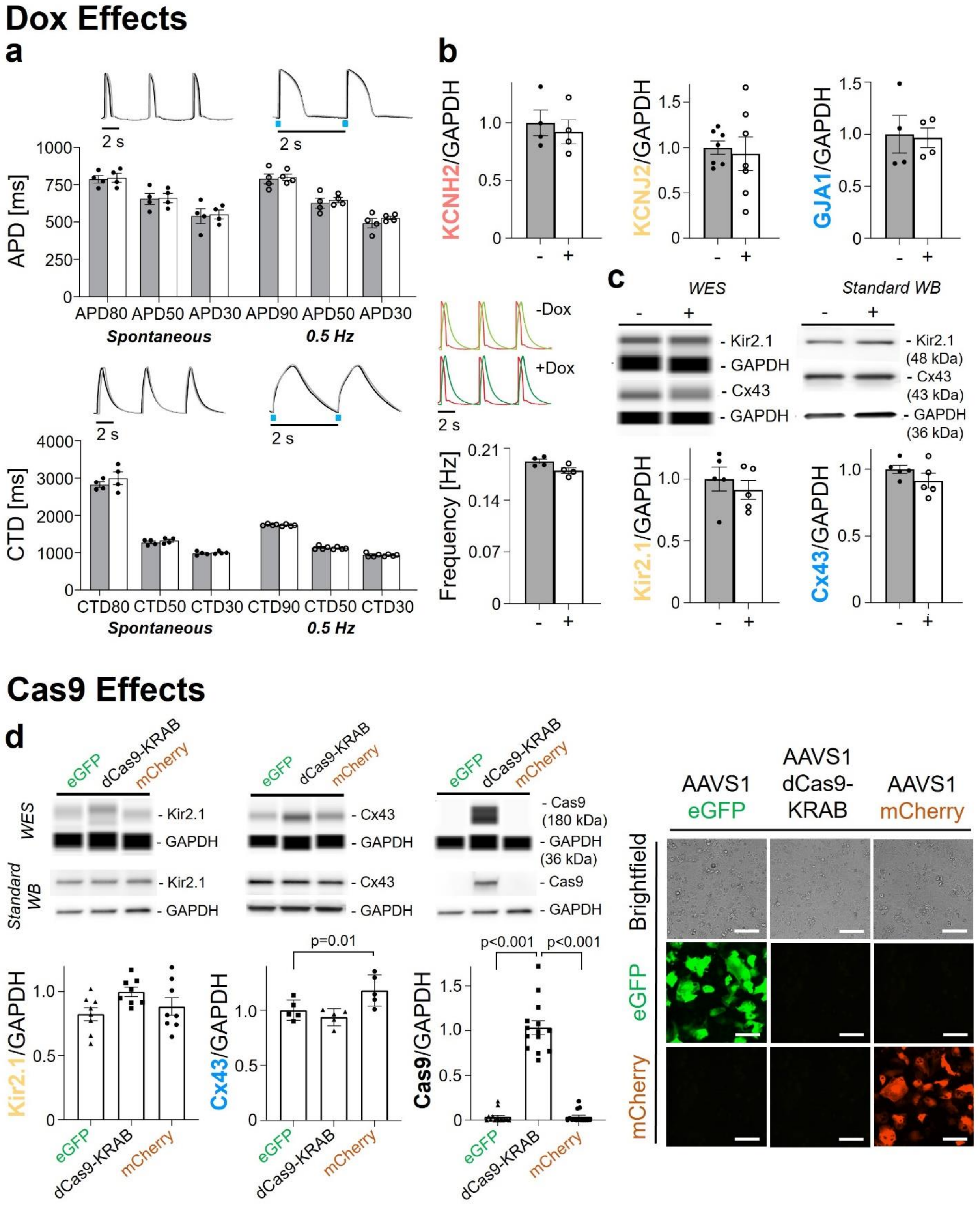

Supplementary Figure 1. Quantification of Dox and dCas9-KRAB effects iPSC-CMs on gene expression of ion channels and functional metrics. (**a-d**) Dox effects: **a)** APD and CTD under spontaneous conditions and 0.5Hz pacing upon 5-day Dox (2 μM) treatment in hiPSC-CMs (n=4 biologically independent samples, two-way ANOVA). Quantification of frequency upon 5-day Dox (2 μM)

treatment in hiPSC-CMs (n=4 biologically independent samples; unpaired t-test). Representative voltage (red) and calcium (green) traces. **b)** mRNA quantification of *KCNH2*, *KCNJ2*, and *GJA1* in hiPSC-CMs cultured in Dox (2 μM) for 5 days (n>4 biologically independent samples, n=3 technical replicates; unpaired t-test). **c)** WES (ProteinSimple) and conventional WB of hiPSC-CMs cultured in Dox (2 μM) for 5-days; representative images (WES, n=2 biologically independent samples; WB, n=3 biologically independent samples; unpaired t-test). **d)** dCas9 effects: WES (ProteinSimple) and conventional WB of hiPSC-CMs, expressing eGFP, dCas9-KRAB, and mCherry, with respective plots; representative images (WES, n=2-5 biologically independent samples; WB, n=3-9 biologically independent samples; one-way ANOVA). Data are presented as mean ± SEM. Scale bar 100 μm.

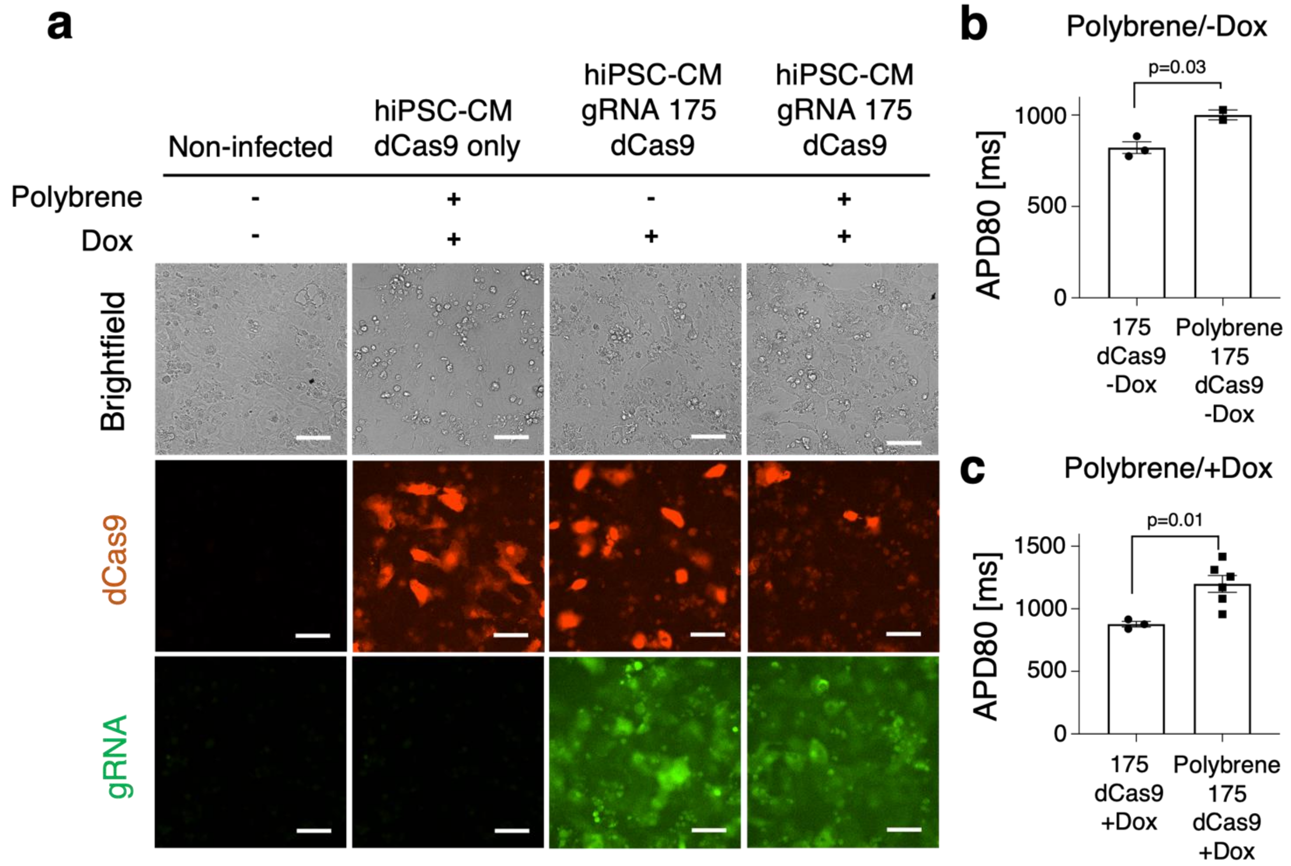

Supplementary Figure 2. Polybrene inhibits transfection post-lentivirus transduction. **a)** Fluorescent images of iPSC-CMs transduced with lentivirus expressing gRNA 175 targeting *KCNH2* with and without Polybrene. Of which Dox-inducible dCas9-KRAB was inserted into the AAVS1 locus. **b)** APD80 of hiPSC-CMs treated with polybrene but no Dox (n>2 biologically independent samples; unpaired t-test). **c)** APD80 of hiPSC-CMs treated with polybrene and Dox (n>3 biologically independent samples; unpaired t-test). Data are presented as mean ± S.E.M. Scale bar 100 μm.

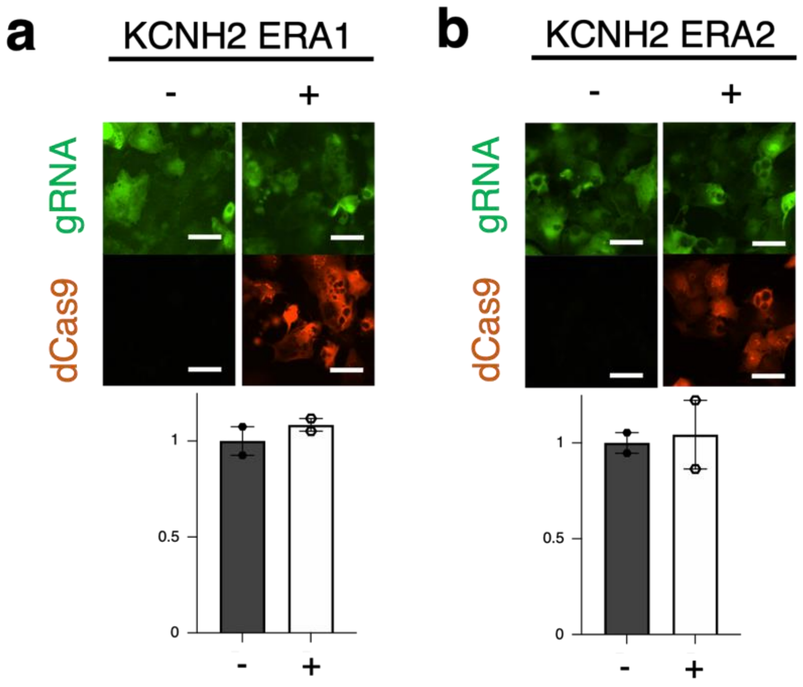

Supplementary Figure 3. Additional gRNA's tested for *KCNH2* knockdown efficiency with *KCNH2* ERA1 and *KCNH2* ERA2. Data are presented as mean ± S.E.M. Scale bar 100 μm.

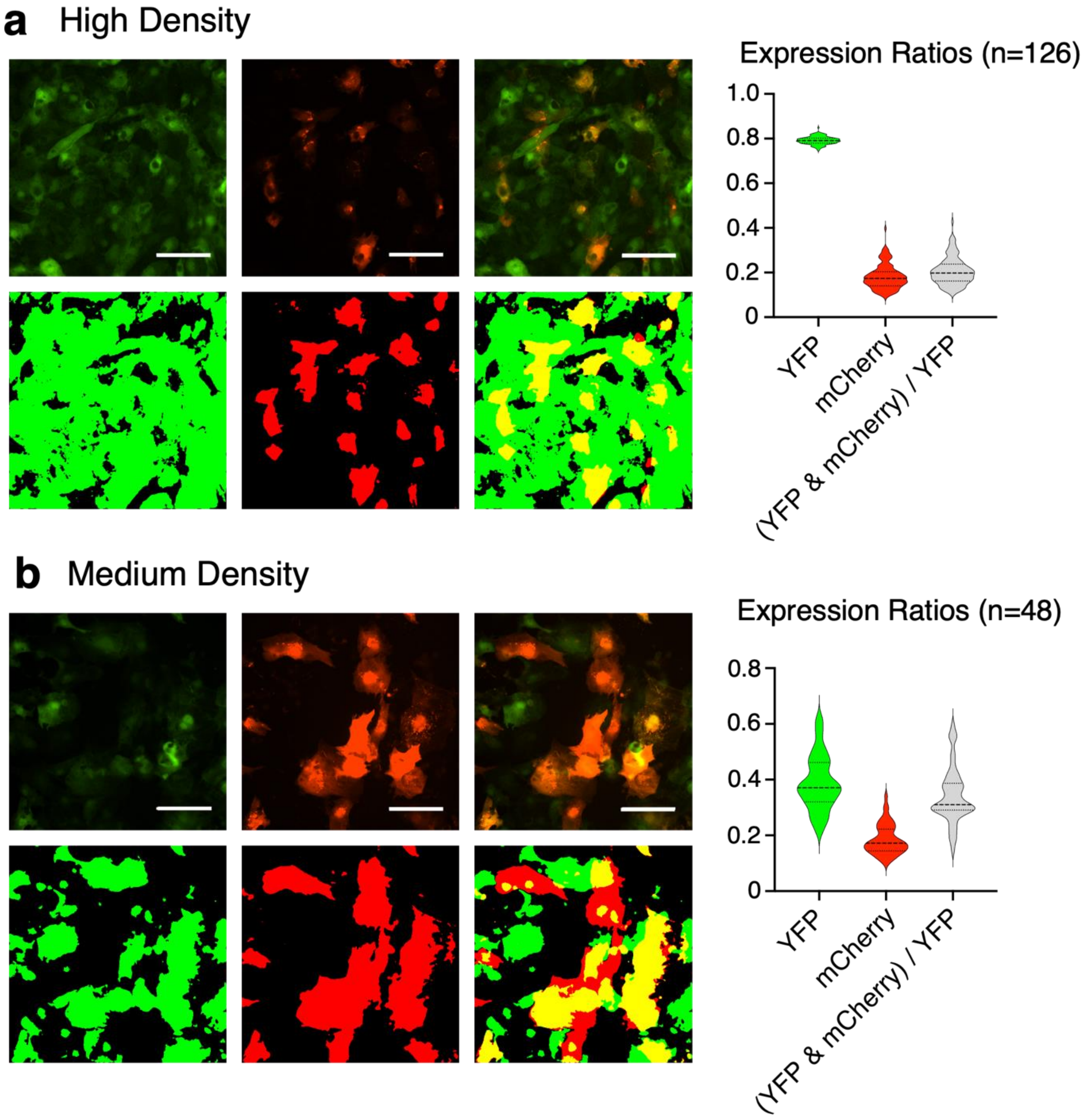

Supplementary Figure 4. Quantification of fluorescence expression ratios of mCherry tagged dCas9-KRAB and eGFP tagged gRNA. **a)** Images of high density groups (n=126 independent ROI), scale bar 100 μm, and medium density groups (n=48 independent ROI), scale bar 50 μm, were analyzed for pixel quantification to calculate regions of overlap in expression of dCas9-KRAB and gRNA. **b)** Plots of expression ratios quantified.

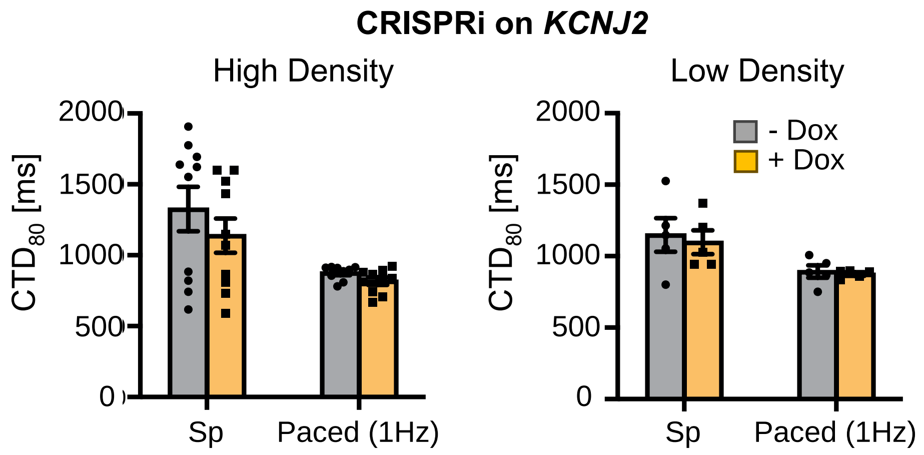

Supplementary Figure 5. Effects of CRISPRi on *KCNJ2* on the duration of the calcium transients at two different plating densities. Spontaneous and paced activity (at 1Hz) are recorded, n = 5–10 biologically independent samples per group. CTD80 is calcium transient duration at 80%. Data are presented as mean ± SEM. No significant differences were detected.

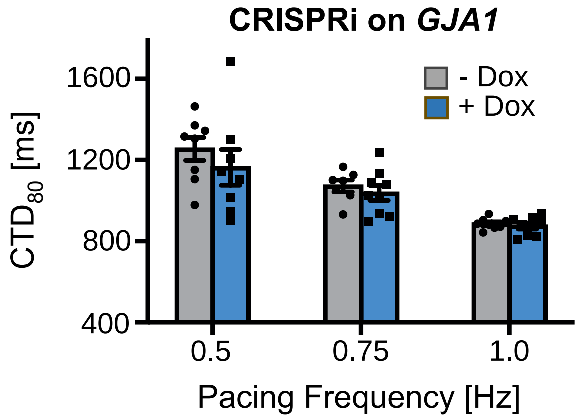

Supplementary Figure 6. Effects of CRISPRi on *GJA1* on the duration of the calcium transients at different pacing rates. Pacing at 0.5Hz, 0.75Hz and at 1Hz was applied, n = 7-9 biologically independent samples per group. Data are presented as mean ± SEM. No significant differences were detected.

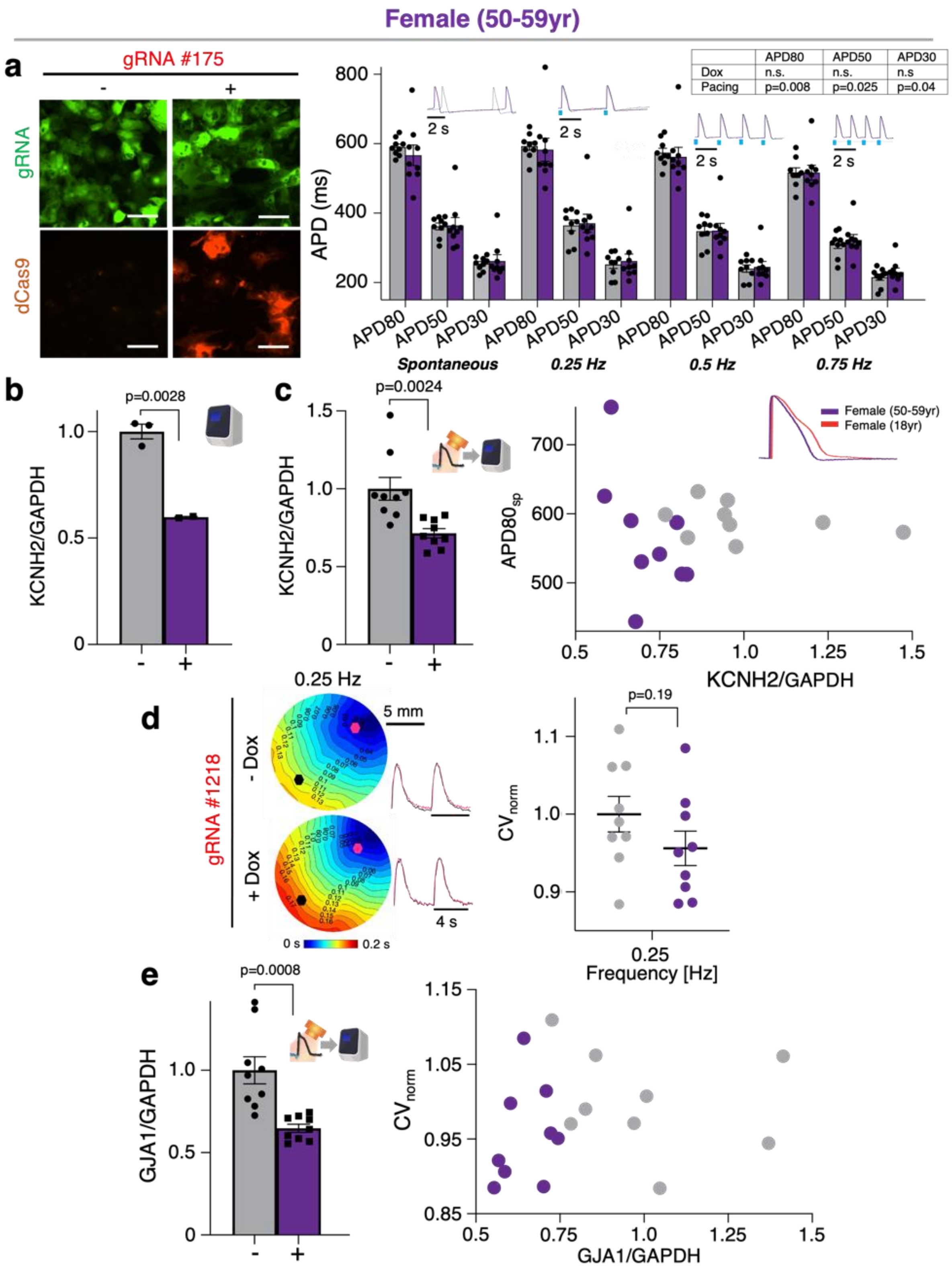

Female (50-59yr)
a
gRNA #175
gRNA
dCas9
APD (ms)
Dox n.s. n.s. n.s
Pacing p=0.008 p=0.025 p=0.04
APD80 APD50 APD30
Spontaneous
0.25 Hz
0.5 Hz
0.75 Hz
2 s
b
p=0.0028
KCNH2/GAPDH
c
p=0.0024
APD80sp
Female (50-59yr)
Female (18yr)
d
0.25 Hz
gRNA #1218
- Dox
+ Dox
5 mm
4 s
0 s
0.2 s
p=0.19
CVnorm
Frequency [Hz]
e
p=0.0008
GJA1/GAPDH

Supplementary Figure 7. CRISPRi modulation of female diversity line. **a)** Fluorescence expression of dCas9-mCherry and gRNA 175 tagged with eGFP upon 5-days Dox (2 μM) treatment in female (50-59yr) hiPSC-CMs; representative images. Functional changes in APD in Dox (2 μM) treated samples expressing Dox-inducible dCas9-KRAB and gRNA targeting *KCNH2* (n=9 biologically independent samples; two-way ANOVA). **b)** qPCR of CRISPRi knockdown of *KCNH2* with gRNA 175 in female hiPSC-CMs (n>2 biologically independent samples, n=3 technical replicates; unpaired t-test). **c)** qPCR analysis of *KCNH2* knockdown post-staining and functional experiments (n=9 biologically independent samples, n=3 technical replicates; unpaired t-test). Plot correlating the effects of relative *KCNH2* mRNA and spontaneous APD80 (n=9 biologically independent samples). **d)** Activation maps of female hiPSC-CMs CRISPRi *GJA1* knockdown and gRNA 1218 at 0.25 Hz pacing; representative images (n=9 biologically independent samples). Conduction velocity changes in Dox (2 μM) treated samples at 0.25 Hz pacing (n=9 biologically independent samples, unpaired t-test). **e)** qPCR of CRISPRi knockdown of *GJA1* with gRNA 1218 post-staining and functional experiments (n=9 biologically independent samples, n=3 technical replicates; unpaired t-test). Plot correlating relative *GJA1* mRNA levels with CV at 1 sec (n=9 biologically independent samples). Data are presented as mean ± S.E.M. Scale bar (100 μm).

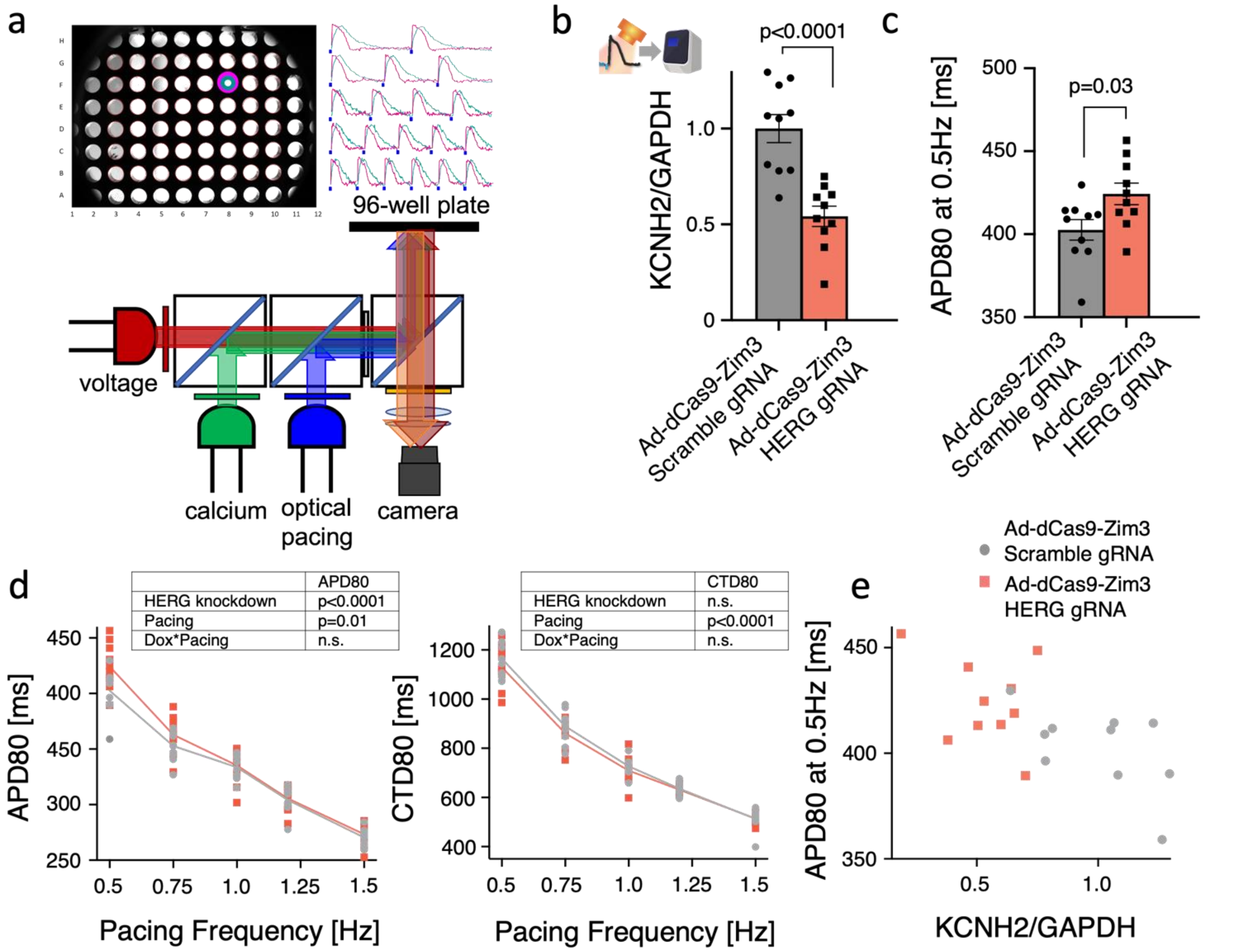

Supplementary Figure 8. Supplementary High-throughput (HT) studies for synchronous, multi-well measurement. **a)** Schematic illustrating optical system capable of simultaneous optical pacing and voltage and calcium imaging of an entire 96-well plate. HT, all-optical electrophysiology studies were conducted at 35°C. **b)** qPCR of dCas9-Zim3 knockdown of *KCNH2* post-staining and functional experiments (n=10 biologically independent samples, n=3 technical replicates; unpaired t-test). **c)** Functional changes in APD80 at 0.5 Hz pacing upon *KCNH2* inhibition by dCas9-Zim3 (n=10 biologically independent samples; unpaired t-test). **d)** Restitution plot of APD80 and CTD80 upon *KCNH2* knockdown (n=10 biologically independent samples; two-way ANOVA). **e)** Plot correlating relative *KCNH2* mRNA levels with APD80 at 0.5 Hz pacing (n=10 biologically independent samples). Data are presented as mean ± S.E.M.

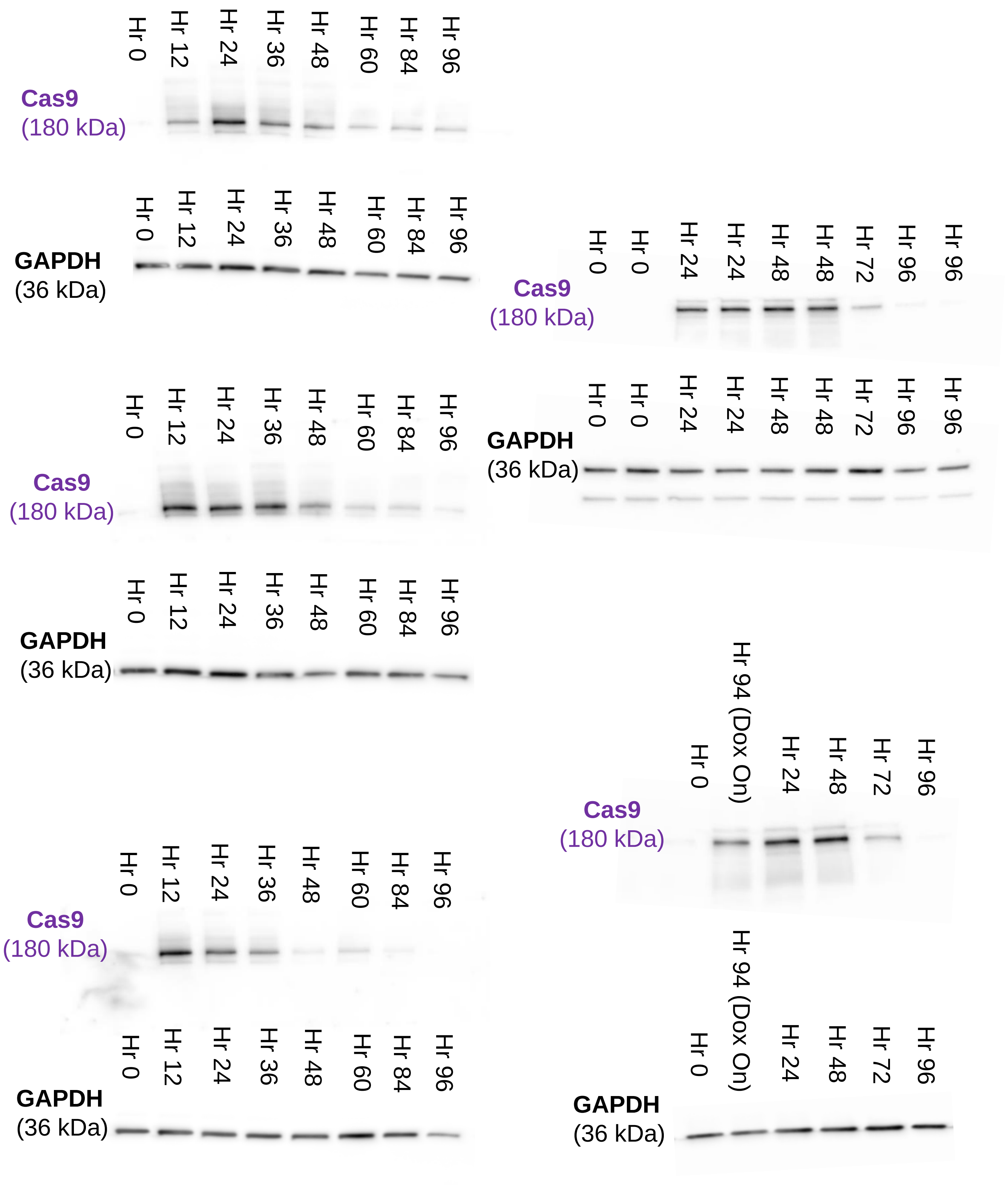

Supplementary Figure 9a. Source blots (standard WB, multiple runs shown) used in Figure 2.

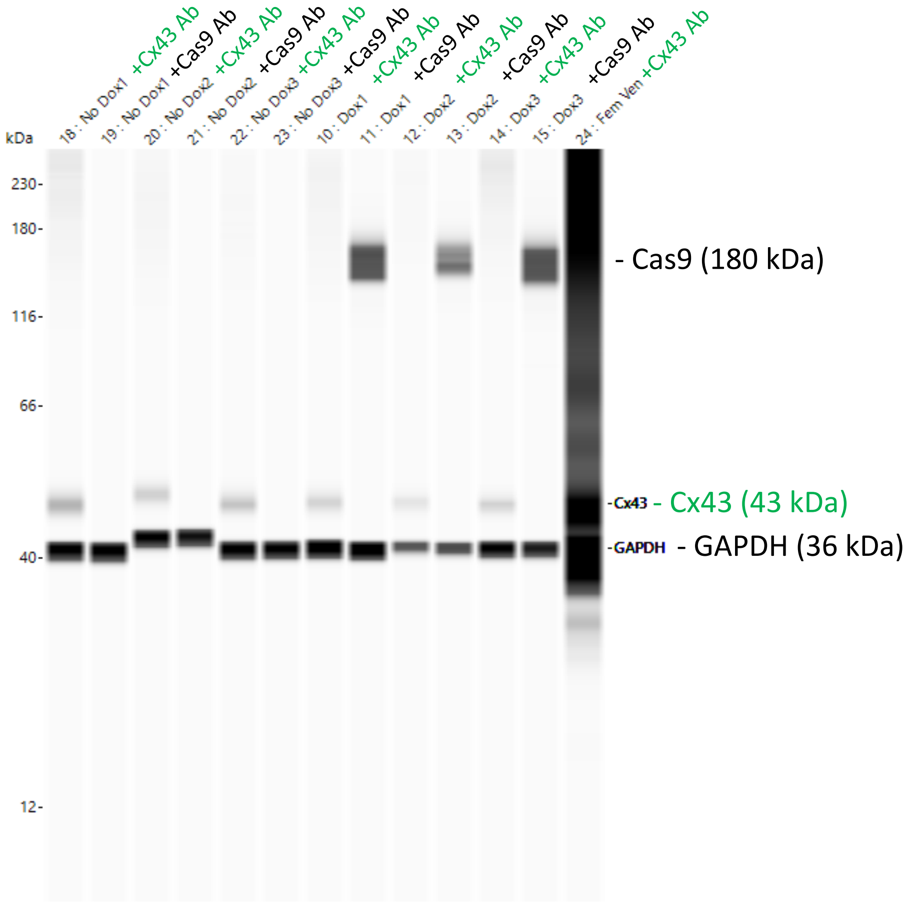

Supplementary Figure 9b. Source WB (Wes) used to plot Figure 5d.

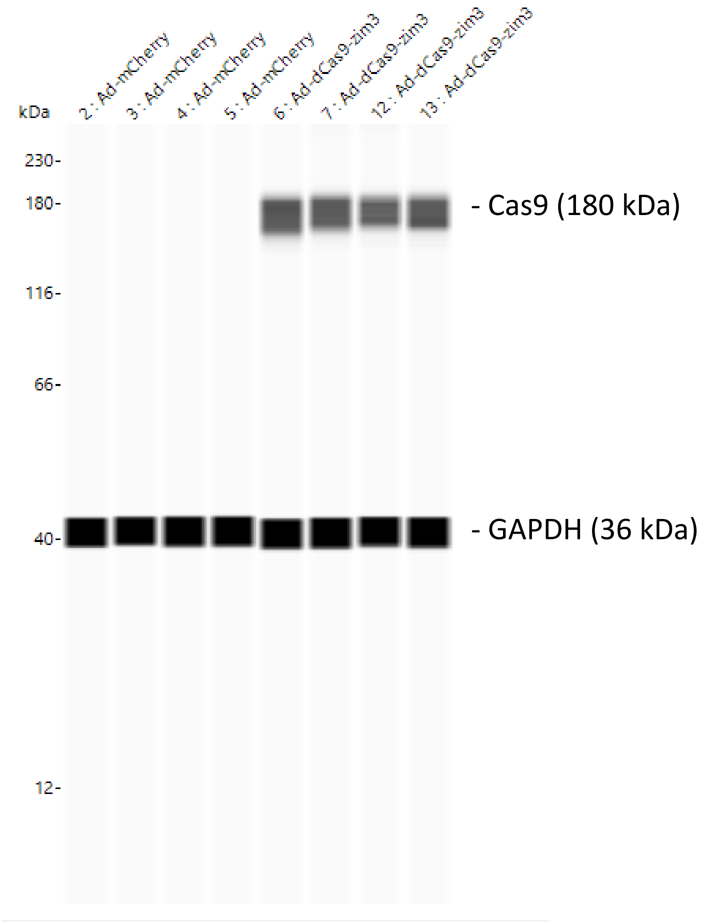

Supplementary Figure 9c. Source WB (Wes) for Figure 7b.

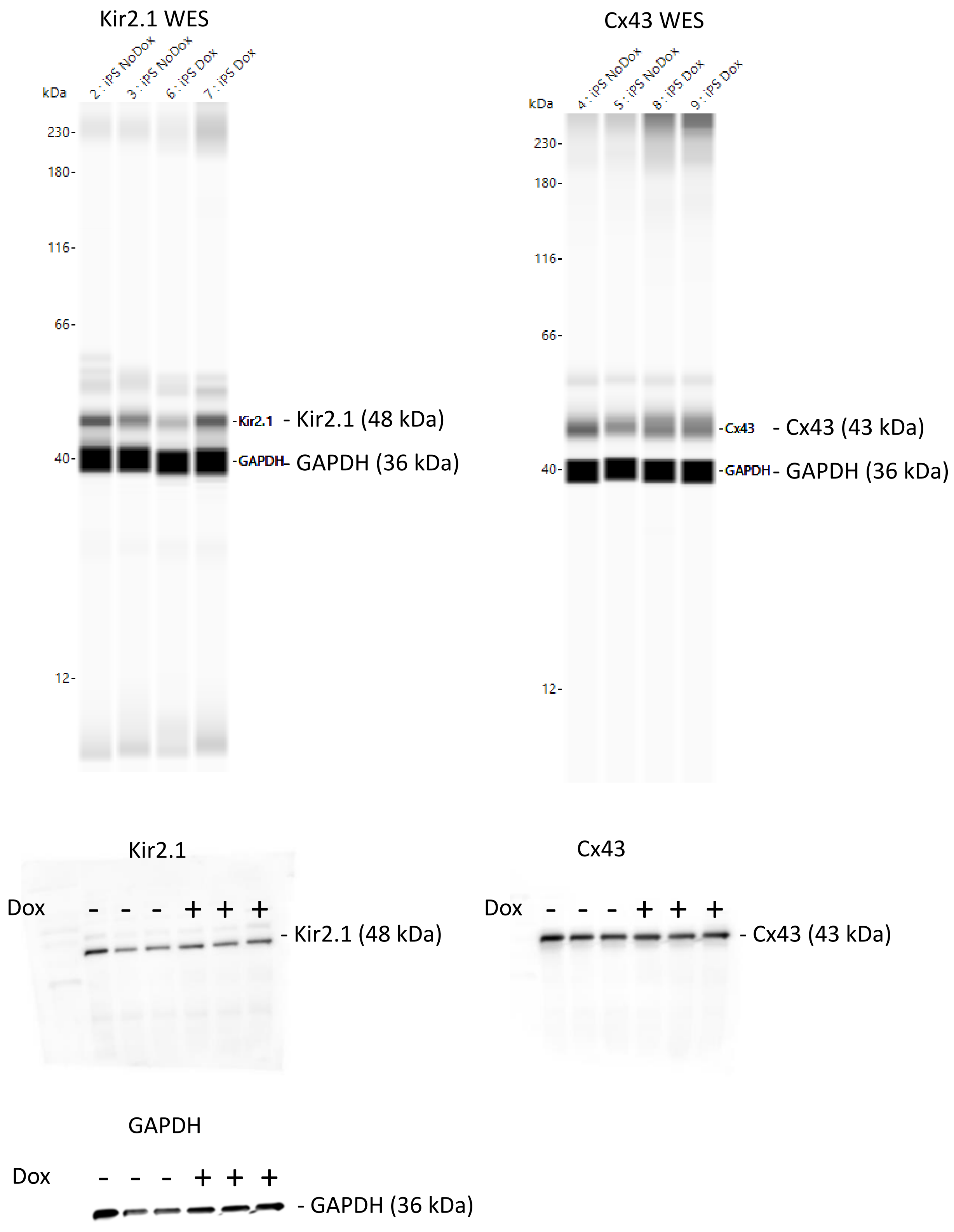

Supplementary Figure 9d. Source blots (Wes and standard WB) for Suppl. Figure 1c.

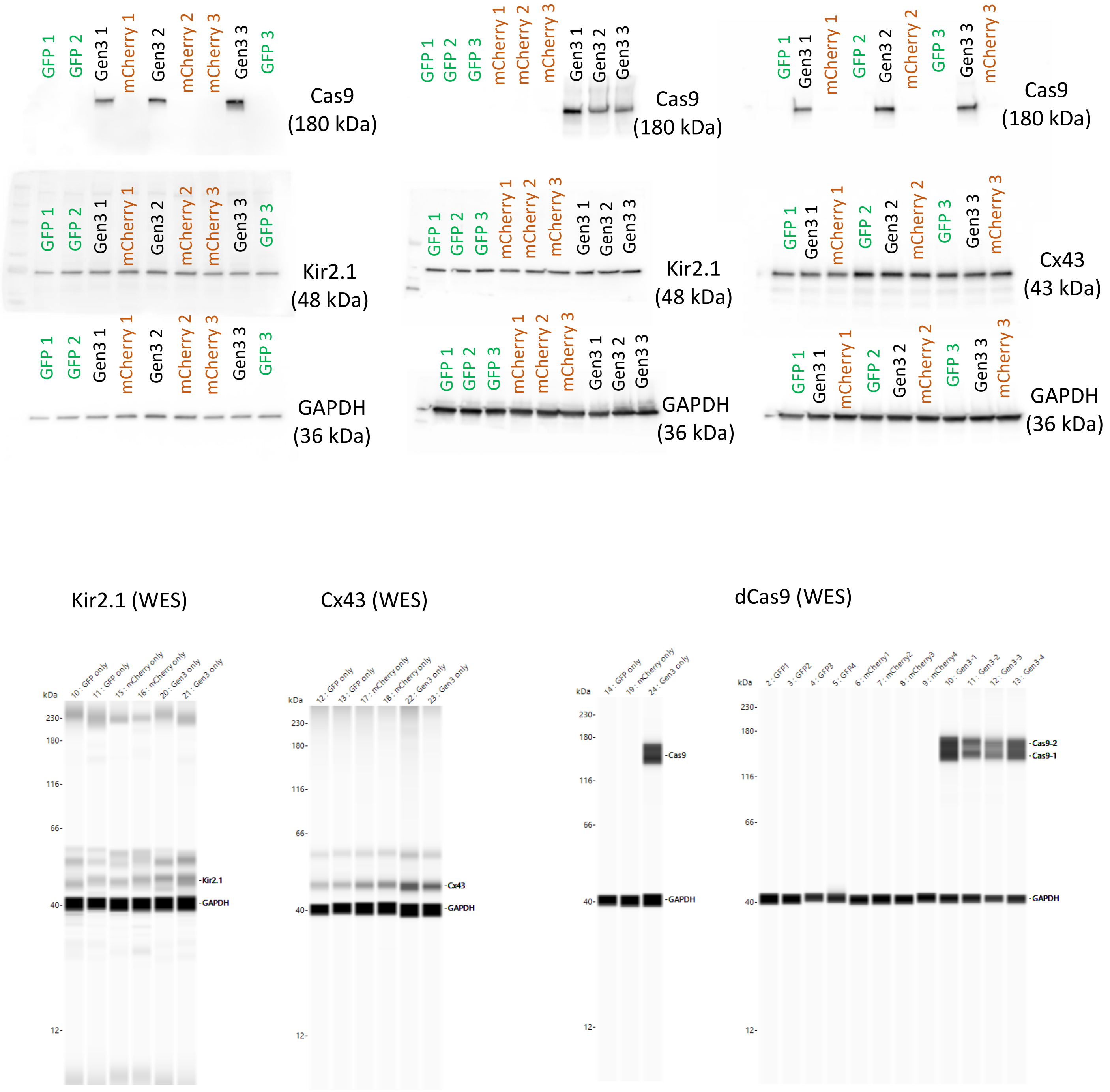

Supplementary Figure 9e. Source blots (Wes and standard WB) for Suppl. Figure 1d.
