# Supplementary material for: CRISPRi gene modulation and all-optical electrophysiology in post-differentiated human iPSC-cardiomyocytes
Source: Commun Biol. 2023 Dec 7;6:1236. doi: 10.1038/s42003-023-05627-y (PMC10703822; doi:10.1038/s42003-023-05627-y)
Supplement: Supplementary file 2 — Description of Additional Supplementary Files [file 42003_2023_5627_MOESM2_ESM.docx]

Description of Additional Supplementary Files

**File name:** Supplementary Data 1

**Description:** All data points used in the Figures

**File name:** Supplementary Data 2

**Description:** Additional data included are fluorescence images for Fig 2, and measured conduction velocity data for Fig. 5d, g and Fig 6d.
