# Supplementary material for: CRISPRi gene modulation and all-optical electrophysiology in post-differentiated human iPSC-cardiomyocytes
Source: Commun Biol. 2023 Dec 7;6:1236. doi: 10.1038/s42003-023-05627-y (PMC10703822; doi:10.1038/s42003-023-05627-y)

SUPPLEMENTARY DATA 2

for

CRISPRi Gene Modulation and All-Optical Electrophysiology in Post-Differentiated Human iPSC-Cardiomyocytes

by Julie L. Han[1], Yuli W. Heinson[1], Christianne J. Chua[1], Wei Liu[1], Emilia Entcheva[1]

[1]Department of Biomedical Engineering, The George Washington University, Washington DC 20052, USA

Source Images for Fig. 2

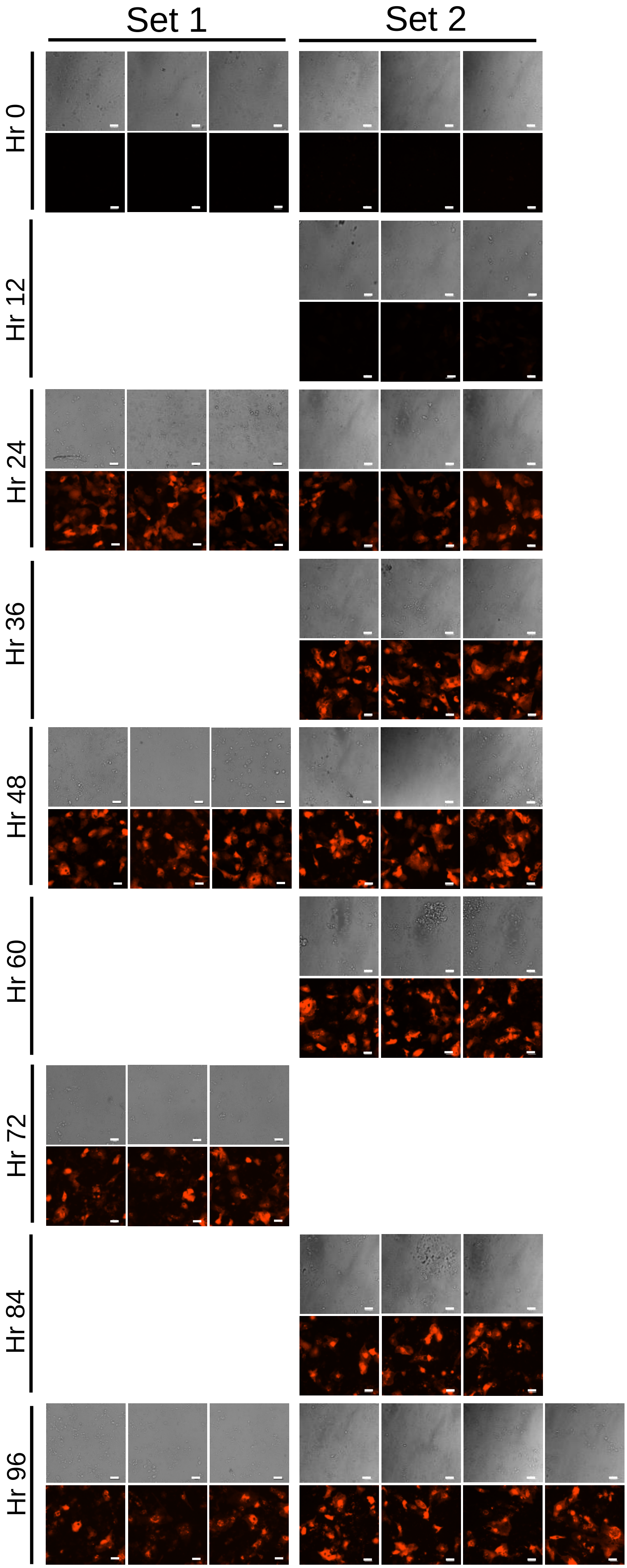

Raw Conduction Velocities for Fig. 5d

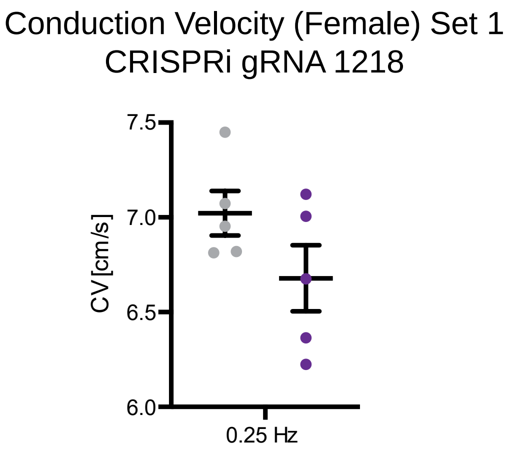

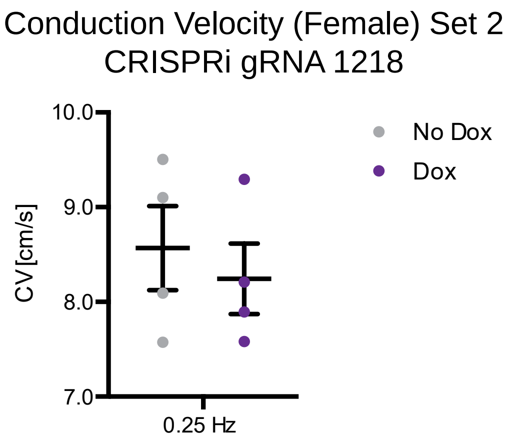

Raw Conduction Velocities for Fig. 5g

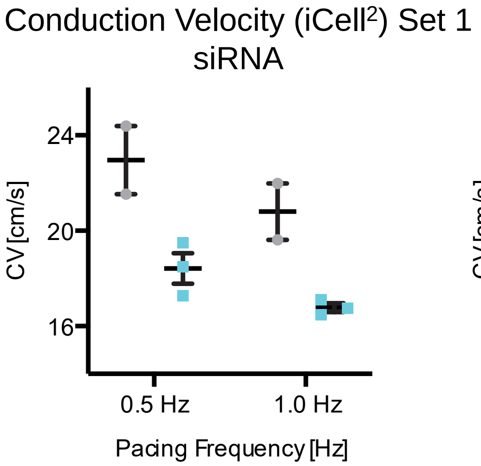

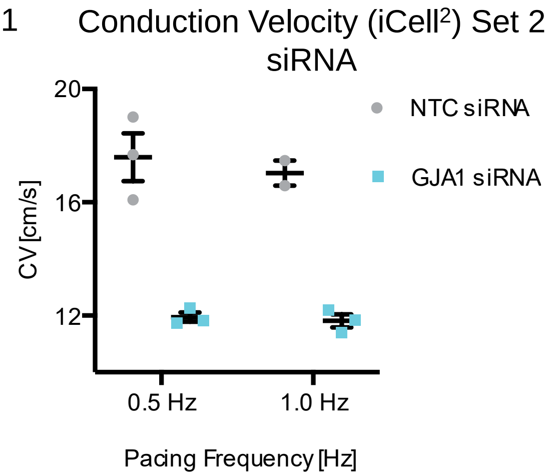

Raw Conduction Velocities for Fig. 6d

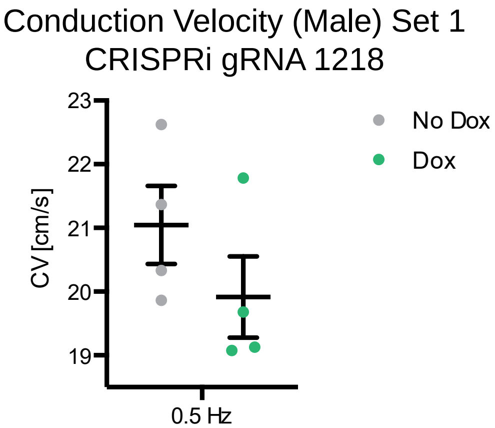

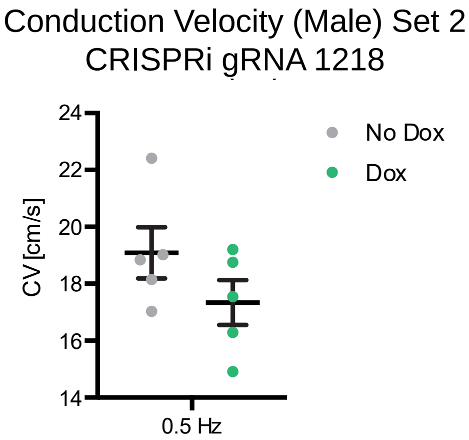

Supplement: Supplementary file 4 — Supplementary Data 2 [file 42003_2023_5627_MOESM4_ESM.pdf]
